# *β-arrestin1*-mediated acetylation of Gli1 regulates Hedgehog/Gli signaling and modulates self-renewal of SHH medulloblastoma cancer stem cells

**DOI:** 10.1186/s12885-017-3477-0

**Published:** 2017-07-17

**Authors:** Evelina Miele, Agnese Po, Federica Begalli, Laura Antonucci, Angela Mastronuzzi, Carlo Efisio Marras, Andrea Carai, Danilo Cucchi, Luana Abballe, Zein Mersini Besharat, Giuseppina Catanzaro, Paola Infante, Lucia Di Marcotullio, Gianluca Canettieri, Enrico De Smaele, Isabella Screpanti, Franco Locatelli, Elisabetta Ferretti

**Affiliations:** 1 Center for Life NanoScience@Sapienza, Istituto Italiano di Tecnologia, 00161 Rome, Italy; 2 Department of Hematology/Oncology and Stem Cell Transplantation, Bambino Gesù Children’s Hospital, IRCCS, 00165 Rome, Italy; 3 Department of Molecular Medicine Sapienza University, 00161 Rome, Italy; 4 Department of Neuroscience and Neurorehabilitation, Neurosurgery Unit, Bambino Gesù Children’s Hospital, IRCCS, 00165 Rome, Italy; 5 Department of Experimental Medicine Sapienza University, Viale Regina Elena, 291 - 00161, 00161 Rome, Italy; 6 Neuromed Institute, 86077 Pozzilli, Italy; 7 Department of Pediatric Science, University of Pavia, Pavia, Italy

**Keywords:** CSCs, Medulloblastoma, Arrb1, Gli1 acetylation, miR-326, Hh/Gli signaling

## Abstract

**Background:**

Aberrant Sonic Hedgehog/Gli (Hh/Gli) signaling pathway is a critical regulator of Sonic hedgehog medulloblastoma (SHH-MB). Cancer stem cells (CSCs), thought to be largely responsible for tumor initiation, maintenance, dissemination and relapse, have been identified in SHH-MB. Since we previously demonstrated that Hh/Gli signaling controls CSCs features in SHH-MB and that in these tumors miR-326 is down regulated, here we investigated whether there is a functional link between Hh/Gli signaling and miR-326.

**Methods:**

We evaluated *β-arrestin1 (Arrb1)* and its intragenic miR-326 levels in CSCs derived from SHH-MB. Subsequently, we modulated the expression of Arrb1 and miR-326 in CSCs in order to gain insight into their biological role. We also analyzed the mechanism by which Arrb1 and miR-326 control Hh/Gli signaling and self-renewal, using luciferase and protein immunoprecipitation assays.

**Results:**

Low levels of Arrb1 and miR-326 represent a feature of CSCs derived from SHH-MB. We observed that re-expression of Arrb1 and miR-326 inhibits Hh/Gli signaling pathway at multiple levels, which cause impaired proliferation and self-renewal, accompanied by down regulation of Nanog levels. In detail, miR-326 negatively regulates two components of the Hh/Gli pathway the receptor Smoothened (Smo) and the transcription factor Gli2, whereas Arrb1 suppresses the transcriptional activity of Gli1, by potentiating its p300-mediated acetylation.

**Conclusions:**

Our results identify a new molecular mechanism involving miR-326 and Arrb1 as regulators of SHH-MB CSCs. Specifically, low levels of Arrb1 and miR-326 trigger and maintain Hh/Gli signaling and self-renewal.

## Background

Tumor mass is composed by heterogeneous cell population including a subset of cells with stem-like characteristics called “cancer stem cells” (CSCs). CSCs could trigger tumor formation, drive resistance to conventional therapeutics and underlie patient relapse [1, 2]. Indeed, stem cell signatures have been associated with poor prognosis in various tumors [1, 3–7]. CSCs have also been identified in medulloblastoma (MB) [8], the most common pediatric malignant brain tumor and a leading cause of cancer-related morbidity and mortality in childhood [9].

Medulloblastoma has been recently classified in 4 molecular subgroups and sonic-hedgehog-driven medulloblastoma (SHH-MB) is the second most common, accounting for 27% of all MBs. They represent an intermediate prognosis subgroup, with overall survival rates ranging from ~35% to ~80% [10]. Recurrence is a common event in SHH-MBs (30%) making the treatment challenging [11].

MB-associated Sonic Hedgehog/Gli (Hh/Gli) pathway deregulation is due to either canonical or non-canonical mechanisms. The canonical Hh/Gli pathway activation is modulated by the receptor Patched (Ptch) that suppresses the activity of Smoothened (Smo) [12, 13]. The binding of SHH protein ligand to Ptch relieves Smo suppression, leading to Hh/Gli activation that culminates in Gli2 transcription factor activation and subsequent translocation to the nucleus [13–15]. Gli2 is able to enhance the transcription of Hh/Gli target genes, including Ptch1 and the transcription factor Gli1, main effector of the signaling. Thus, mutations/focal deletions or amplifications of genes encoding pathway components, such as Ptch1, Smo, and Gli2 are well-recognized oncogenic events in SHH-MBs. On the other hand, non-canonical Hh/Gli activation mechanisms have been described, involving post-transcriptional modification of Gli1, histone methylation, p53/17p deletion and PI3K/Akt/S6 K aberrant activation [9, 16–22].

microRNAs (miRNAs) are major regulators of Hh/Gli signaling [23] and we have previously shown that miR-326 is downregulated in SHH-MBs where it inhibits Smo [23]. Recent evidence highlighted the crucial role of miRNAs also in CSCs [24]. The pivotal role of Hh/Gli pathway in controlling CSCs maintenance, including SHH-MB, has already been demonstrated [25–28].

We have previously isolated and characterized MB CSCs from mouse model of SHH-MB [26]. Such CSCs were capable to grow as oncospheres in stem cell-medium and expressed the stemness marker Nanog under Hh/Gli transcriptional regulation [26].

Since a thorough understanding of the molecular mechanisms that govern the maintenance of CSCs is necessary to unveil SHH-MB biology/behavior we decided to further investigate the Hh/Gli-miR-326 network in SHH-MB CSCs context.

Here we show that miR-326 and its host gene Arrb1 are both down regulated in CSCs derived from SHH-MB, where they act as negative regulators of self-renewal. Indeed, their expression inhibits Hh/Gli signaling at multiple levels: Arrb1 potentiates p300-mediated Gli1 inhibitory acetylation and miR-326 targets Smo and Gli2.

## Methods

### Animals

Murine CSCs were isolated, as previously reported [26] from Ptch1+/− mice model of SHH-MB (The Jackson Laboratory, Bar Harbor, ME, USA) maintained in the Molecular Medicine Department Animal Facility at Sapienza University of Rome. Experiments were carried out on CSCs derived from 6 different Ptch1+/− mice. All experiments were performed in accordance with national guidelines and regulations, and with the approval of the animal care and use committees of our institution.

### CSC cultures, oncosphere-forming assay, differentiation, over-expression, silencing and proliferation assay

CSCs were cultured as previously reported [26]. Selective medium (SM) was used for CSCs enrichment, consisting of DMEM/F12 with B27 supplement without vitamin A and 2 mg/ml heparin, 0,6% glucose, 60 mg/ml N-acetyl-L-cysteine, 25 μg/ml insulin, 20 ng/ml EGF, 20 ng/ml bFGF.

Oncosphere-forming assay was performed as previously described [26]. In detail, cells were plated at clonal density (1–2 cells/mm^2^) into 96-well plates and cultured in SM.

To induce differentiation, oncospheres were mechanically dissociated and plated into D-poly-lysine coated dishes in differentiation medium (DFM): DMEM/F12 with N2 supplement and 2 mg/ml heparin, 0,6% glucose, 60 mg/ml N-acetyl-L-cysteine, 1% Calf Serum and retinoic acid 2 μM.

Amaxa nucleofector (Lonza) was used to transfect plasmids according to manufacturer’s procedure. miR-326 vector and its negative control were purchased from GeneCopoeia (MmiR3333-MR01); Arrb1 vector was obtained from Addgene [29]. For rescue experiments, cells were transfected with both miR-326 vector and SmoM2 and Gli2-Flag plasmid vectors [23, 26].

Silencing of Arrb1 was performed with HiPerFect (Qiagen) using ON-TARGETplus SMARTpool (L40976–00-005 mouse ARRB1) from Thermo Scientific, after testing each single siRNA of the pool, alone or in combination, for its specificity to avoid OFF-target effects.

Proliferation of MB CSCs was evaluated by BrdU incorporation, as previously described [30]. Cells were counted in triplicate and the number of BrdU-positive nuclei was annotated. MB CSCs growth was measured by MTS (Promega) assay according to manufacturer’s instructions. Each sample was measured in triplicate and repeated at least three times.

HEK293T cells were cultured and transfected as previsouly described [26] with the indicated plasmids as in [31].

### Western blot and immunoprecipitation assays

Cells were lysed using RIPA buffer (Tris-HCl pH 7.6 50 mM, deoxycholic acid sodium salt 0.5%, NaCl 140 mM, NP40 1%, EDTA 5 mM, NaF 100 mM, sodium pyrophosphate 2 mM and protease inhibitors). Lysates were separated on 8% acrylamide gel and immunoblotted using standard procedures. The following antibodies were used: anti-Arrb1 K-16 (sc-8182; Santa Cruz Biotechnology), anti-Nanog (Cosmo Bio Co, Japan), anti-Actin I-19 (sc-1616; Santa Cruz Biotechnology), anti-β-III-Tubulin (MAB 1637 Millipore), anti-Gli1 H-300 (sc-20,687; Santa Cruz Biotechnology), anti-acetyl-Gli1 (Lys518) (Eurogentec) [32], anti-p300 C-20 (sc-585; Santa Cruz Biotechnology), anti-FLAG M2-Peroxidase (HRP) (A8592 Sigma), anti-HA (sc-7392 Santa Cruz), anti-Gli2 H-300 (sc-28,674; Santa Cruz Biotechnology), anti-Smo N-19 (sc-6366; Santa Cruz Biotechnology), anti-Sox2 (MAB4343 Millipore). HRP-conjugated secondary antibodies (Santa Cruz Biotechnology) were used in combination with enhanced chemo-luminescence (ECL Amersham).

For immunoprecipitation assay antibody sources and concentrations used were: Protein G Plus-Agarose (sc-2002; Santa Cruz Biotechnology); anti-FLAG M2 Affinity Gel (Sigma A2220, IP 30⌠l), anti-FLAG M2-Peroxidase (HRP) (A8592 Sigma, western blotting 1:5000), anti-HA (sc-7392 Santa Cruz, 1:1000); anti-myc-HRP.

### Immunofluorescence

CSCs were plated on D-poly-lysine-coated Lab-Tek chamber slides (coverslips) and allowed to adhere for 3 h. For the staining of differentiated cells, cells were cultured on D-poly-lysine-coated coverslips in DFM for 48 h. Cells were fixed with 4% paraformaldehyde for 20 min at room temperature, incubated in blocking solution (5% normal goat serum (NGS), 1% BSA, 0.1% Triton X-100) and stained overnight with primary antibodies diluted in blocking solution and 2 h with secondary antibodies. Primary antibodies were anti-Nanog (Cosmo Bio Co, Japan), anti-Nestin (AB6142, Abcam) and anti-Gli1 (#2643 Cell Signaling Technology Inc); 488-conjugated anti-mouse and anti-rabbit secondary antibodies were purchased from Molecular Probes (Invitrogen, Eugene, OR). Nuclei were counterstained with Hoechst reagent. Cover slips were mounted with fluorescence mounting medium (S3023, Dako). Images were acquired with Carl Zeiss microscope (Axio Observer Z1) using Apotome technology and AxioVision Digital Image Processing Software.

### RNA isolation and qRT-PCR

Unless otherwise indicated, reagents and equipment were purchased from Thermo Fisher Scientific. Total RNA was purified using Trizol and treated with DNase. One μg was reverse transcribed using random primers and SuperScript II as previously described [23]. Quantitative RT-PCR (qRT-PCR) analysis was performed using the ABI Prism 7900HT Sequence Detection System, using the “best coverage” TaqMan gene expression assays, specific for each analyzed mRNA, according to manufacturer’s protocol. Each amplification reaction was performed in triplicate, and the average of the three threshold cycles was used to calculate the amount of transcripts in the sample (SDS 2.3 software). mRNA quantification was expressed, in arbitrary units, as the ratio of the sample quantity to the calibrator or to the mean values of control samples. All data were normalized to the mean value of three endogenous controls: GusB, β2-microglobulin and HPRT.

miR-326 expression was normalized to RNU6B: both were measured using TaqMan microRNA assays according to manufacturer’s instructions.

### Luciferase and mutagenesis assays

The putative miR-326 binding site on Gli2 3’UTR was identified by bioinformatics analysis using the combination between miRanda and Target Scan algorithm (http://www.microrna.org/microrna/home.do). The entire 3’UTR region of mouse Gli2 was purchased from GeneCopoeia in pEZX-MT01 vector (MmiT025993-MT01). This construct was used to obtain the mutant derivate lacking the entire miR-326 binding sequence, using the QuickChange XL Site-Directed Mutagenesis kit (Agilent Technologies). MB CSCs were transfected with 3’UTR plasmids of wild type Gli2–3’UTR or mutant Gli2–3’UTR and miR-326 vector or the empty control with Fugene6 Transfection Reagent (Promega). For analysis of luciferase activity from the Gli-responsive reporter in presence of Arrb1, cells were transfected with Gli-responsive reporter (Gli8x_luc) and with a wild-type Gli1 vector (Gli1 wt) or a Gli1 mutant (Gli1 K518R) [20], together with the Arrb1 plasmid or an empty vector as control. In all luciferase experiments pRL-CMV-Renilla Luciferase control vector was used. After 24 h cells were collected and tested with dual luciferase-assay (Promega). All luciferase activity data are presented as mean ± S.D. of values from at least three experiments in triplicate.

For mutagenesis of miR-326 binding site on Gli2–3’UTR, the following primers were used:

- Fw: CCCAGGGCAGCAAACTCAGGACCAACTCCAAA
- Rw: TTTGGAGTTGGTCCTGAGTTTGCTGCCCTGGG

### SHH-MB samples

Surgical specimens used in this study originate from a cohort of patients, recruited with Institutional Review Board approval of the contributing Centers, as previously described [23, 33]. For this study 10 ng of cDNA from each MB was analyzed for the expression levels of genes specific for SHH-MB molecular classification (as described in [10, 34]).

In detail for ARRB1 and pri-miR-326 expression analysis *n* = 17 cDNA of human SHH MBs and 10 adult cerebella as control were evaluated by RT-PCR and statistical differences were assessed by Mann–Whitney *U* test for non-parametric values using GraphPad Prism 6 software. Regression analysis was performed using GraphPad Prism 6 software.

### Statistical analysis

Statistical analysis of cellular experimental triplicates was performed using StatView 4.1 software (Abacus Concepts, Berkeley, CA). Statistical differences were analysed by Mann–Whitney *U* test for non-parametric values and a *p*-value of 0.05 was considered significant. The results are expressed as mean ± S.D. from an appropriate number of experiments as indicated in the respective figure legends.

## Results

### *Low levels of miR-326 characterize* SHH-MB CSCs

**Fig. 1:**
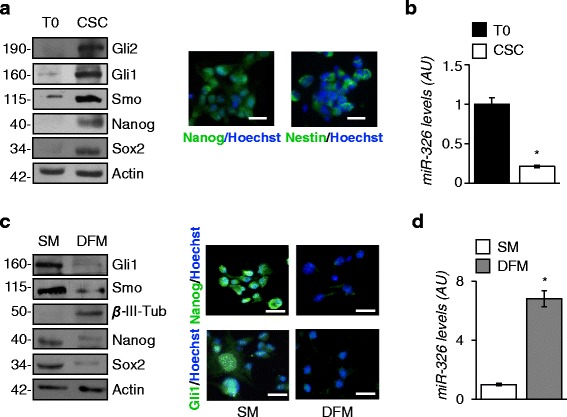
*Low levels of miR-326 characterize SHH-MB CSCs.* **a** *Left*: Western blot (WB) analysis of endogenous Hh/Gli and stemness markers levels in pre-oncosphere cell population (T0) and oncospheres (CSCs) derived Ptch1+/− mice model of SHH-MB. Loading control (LC): Actin. Right: Immunofluorescence of nuclear Hoechst (*blue*) staining with Nanog or Nestin (*green*) in CSCs. Scale bar: 10 ⌠m. **b** miR-326 expression levels in MB cells grown in SM (CSCs) vs pre-oncosphere cell population (T0) are expressed in arbitrary units (AU). Bar graphs represent mean ± S.D. from three independent experiments. *p < 0.05. **c** *Left panel*: WB analysis of endogenous Hh/Gli, stemness and differentiation markers levels in CSCs grown as oncospheres in SM and exposed to differentiation stimuli (DFM). LC: Actin. Right panel: Immunofluorescence of nuclear Hoechst (blue) staining with Nanog or Gli1 (*green*) in CSCs grown in SM or DFM. Scale bar: 10 μm. **d** miR-326 expression levels in MB cells grown in SM (CSCs) or in DFM

We have previously identified miR-326 as a negative regulator of Hh/Gli signaling in cerebellar granule cell progenitors (GCPs) (23). Next from SHH-MB of Ptch1 +/− mice [28], we isolated and characterized CSCs capable to grow as oncospheres in stem cell-medium [26]. In this study we aimed to investigate the Hh/Gli signaling and miR-326 network in SHH-MB CSCs context. First we observed that Hh/Gli signaling components, including Gli1, Gli2 and Smo, together with stemness markers Nanog, Sox2 and Nestin were enriched in CSCs respect to SHH-MB Ptch1 pre-oncosphere cell populations (T0) (Fig. 1a). CSCs were also characterized by low levels of miR-326 in respect to T0 (Fig. 1b). When shifted to differentiation medium (DFM), CSCs expressed the neuronal marker β-III-tubulin and down regulated the expression of Hh/Gli pathway components and stemness markers (Fig. 1c) while miR-326 expression increased (Fig. 1d).

Overall our findings showed that miR-326 is inversely expressed respect to Hh/Gli signaling and stemness in SHH-MB CSCs.

### *Arrb1 is down regulated in* SHH-MB CSCs

**Fig. 2:**
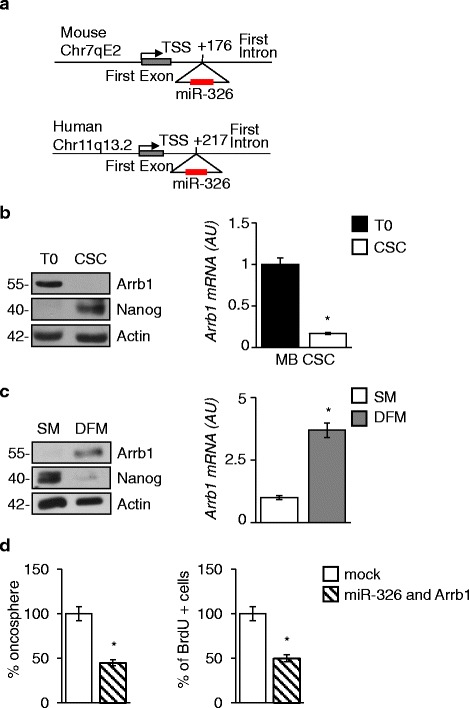
*Arrb1 is down regulated in SHH-MB CSCs.* **a** miR-326 intragenic localization in the first intron of the Arrb1 gene on murine chromosome 7 and human chromosome 11. **b** WB (*Left panel*) of Arrb1 and Nanog levels in SM (CSCs) vs pre-oncosphere cell population (T0). mRNA expression (*Right panel*) analysis of Arrb1 (Arrb1) levels MB cells grown in CSCs vs T0. Bar graphs represent mean ± S.D. from three independent experiments. *p < 0.05. **c** Arrb1 and Nanog expression levels in CSCs grown as oncospheres in SM and exposed to differentiation stimuli (DFM). LC: Actin. **d** Oncosphere forming assay (*left panel*) and bromodeoxyuridine (BrdU) uptake (*right panel*) in CSCs after ectopic expression of miR-326 and Arrb1. Data represent mean ± S.D. from five independent experiments. *p < 0.05

miR-326 coding gene resides in the first intron of the host gene *Arrb1* in mouse chromosome 7q and in human chromosome 11q (Fig. 2a). Notably, miR-326 and *Arrb1* share common regulatory sequences acting as a single transcriptional unit [35]. Together these data prompted us to investigate *Arrb1* in CSCs. We found low Arrb1 expression levels of both mRNA and protein (Fig. 2b), while Arrb1 was expressed in differentiated CSCs (DFM) (Fig. 2c). These results highlighted that low levels of the transcription unit *Arrb1 and miR-326* are associated with SHH-MB CSCs. The ectopic re-expression of Arrb1 and miR-326 impaired their clonogenic potential (expressed as the percentage of oncospheres formation) and their proliferation rate (Fig. 2d).

These results support a role for Arrb1 and miR-326 in the establishment and maintenance of a “differentiated cell-phenotype”.

### miR-326 and Arrb1 impair stemness through suppression of Hh/Gli pathway at multiple levels.

The observation of low levels of miR-326 and Arrb1 in CSCs derived from SHH-MBs suggests that they can negatively regulate the major pro-proliferative signaling in these cells, namely the Hh/Gli pathway [25–28].

**Fig. 3:**
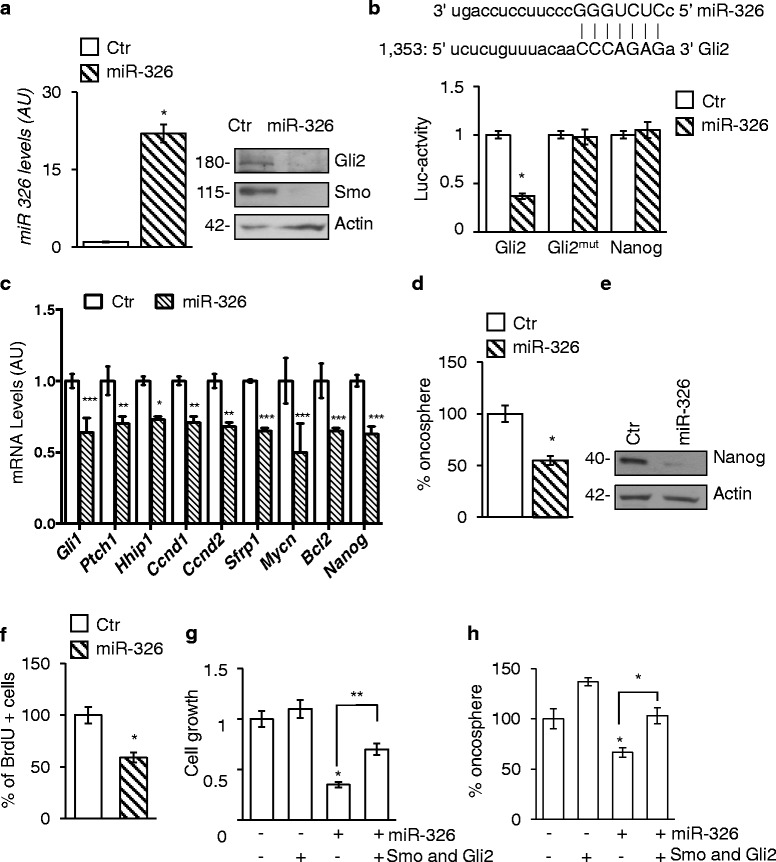
*miR-326 inhibits stemness by targeting Hh/Gli signaling.* **a** miR-326 levels (*left panel*) and Gli2 and Smo western blot analysis (*right panel*) in CSCs overexpressing miR-326. *p < 0.05. LC: Actin. **b** *Upper panel*: putative miR-326 binding site on the Gli2 3’UTR (miRanda algorithm). *Lower panel*: luciferase activity in CSCs overexpressing miR-326 and transfected with either the Gli2 wild type 3’UTR vector (Gli2) or the Gli2mut derivative, lacking the miR-326 binding site. Nanog 3’UTR vector (no putative miR-326 binding sites) as negative control. Results are expressed as a ratio vs scrambled miRNA vector-transfected cells (Ctr). Data represent mean ± S.D. from three independent experiments. *p < 0.05. **c** Histograms showing mRNA expression levels of the indicated Hh target genes in CSCs overexpressing miR-326 compared to control empty vector (Ctr). Data represent mean ± S.D. from three independent experiments. *p < 0.05; **p < 0.01; ***p < 0.005. **d** Oncosphere forming assay in CSCs after ectopic expression of miR-326. Data represent mean ± S.D. from three independent experiments. *p < 0.05. **e** WB analysis of endogenous Nanog in CSCs overexpressing miR-326 or scramble miRNA as control (Ctr). LC: actin. **f** BrdU uptake in MB CSCs after ectopic expression of miR-326. Data represent mean ± S.D. from three independent experiments. *p < 0.05. **g** Cell growth assessed through MTT assay in CSCs overexpressing miR-326 together or not with the co-expression of SmoM2 and Gli2-Flag (Smo and Gli2) plasmid vectors. Data represent mean ± S.D. from three independent experiments. *p < 0.05. **h** Oncosphere forming assay in CSCs overexpressing miR-326 together or not with the co-expression of SmoM2 and Gli2-Flag (Smo and Gli2) plasmid vectors. Data represent mean ± S.D. from three independent experiments. *p < 0.05

Here we showed that miR-326 re-expression in SHH-MB CSCs inhibited the Hh/Gli signaling at both receptor and transcription factor levels. Indeed, miR-326 reduced Smo protein, as already described in undifferentiated GCPs [23], but also downregulated Gli2 levels (Fig. 3a). In silico analysis revealed the presence of putative miR-326 binding sites in the Gli2–3‘UTR (Fig. 3b *upper panel*). miR-326 overexpression repressed the activity of a reporter construct carrying the mouse Gli2–3’UTR (Fig. 3b, *bottom panel*) but had no effect on either Gli2–3’UTR construct with mutated miR-326-binding sites or on the unrelated Nanog-3’UTR reporter (Fig. 3b, *bottom panel*). Consistent with an inhibitory role of miR-326 on Hh/Gli signaling we observed that the overexpression of miR-326 impaired the expression levels of the transcription target genes of the Hh/Gli pathway as defined by KEGG pathway analysis and literature *Gli1, Ptch1, Hhip1, Mycn, Ccnd1, Ccnd2, Bcl2, Nanog, Srfp1* [36, 37] (Fig. 3c).

Next, since Hh/Gli pathway controls CSCs [25] and their stemness marker Nanog [26] we investigated whether miR-326 re-expression impairs self-renewal and cell proliferation rate in our cellular model. Indeed, overexpression of miR-326 in SHH-MB CSCs significantly impaired their clonogenic ability (Fig. 3d). These results are consistent with a concomitant suppression of Nanog protein expression (Fig. 3e) and impairment of proliferation rate (Fig. 3f). In accordance with miR-326-mediated targeting of Smo and Gli2, overexpression of plasmid vectors harboring the open reading frame of these genes together with miR-326, rescued the miRNA-induced inhibition of cell growth (Fig. 3g) and clonogenic activity (Fig. 3h). These results highlighted that low miR-326 maintains CSCs features by controlling Hh/Gli signaling components.

Hence we evaluated the function of the host gene of miR-326, Arrb1, in SHH-MB CSCs. *Arrb1* encodes a multifunctional adaptor and scaffold protein regulating several signaling pathways critically involved in cell development in both physiological and pathological (i.e. cancer) contexts [38]. Arrb1 was reported to function as a protein that interacts with the histone acetyl-transferase (HAT) facilitating its recruitment to target histones, with consequent increased chromatin acetylation and transcription activation [31, 39]. We previously reported that Gli transcription factors activity is regulated by acetylation via the acetyl-transferase p300 [20, 32].

**Fig. 4:**
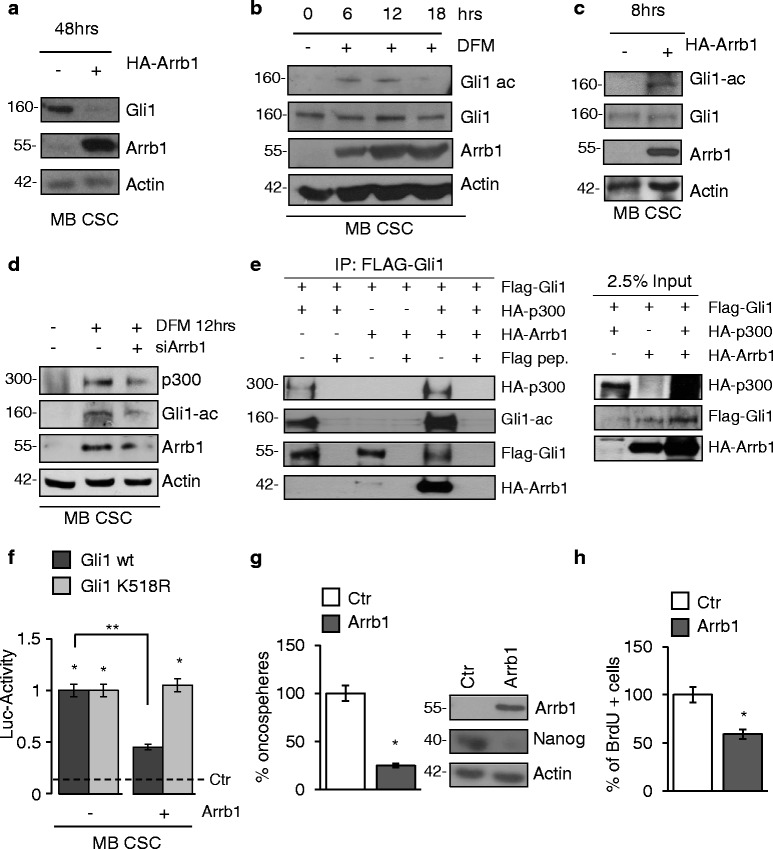
*Arrb1* mediates acetylation of Gli1. **a** Arrb1 and Gli1 protein levels in CSCs transfected with the arrb1-HA plasmid and analyzed 48 h after transfection. LC: Actin. **b** WB analysis of endogenous Arrb1, Gli1 and its acetylated form (Gli1-ac) in CSCs cultured in SM in DFM for the indicated time points. LC: Actin. **c** Arrb1, Gli1 its acetylated form (Gli1-ac) protein levels in CSCs transfected with the arrb1-HA plasmid and analyzed 8 h after transfection. LC: Actin. **d** Arrb1, acetylated Gli1 (Gli1-ac) and p300 protein levels in CSCs transfected with control siRNA (siCtr) or Arrb1 siRNA (siArrb1) cultured for 12 h in DFM. LC: Actin. **e** HEK293T cells were transfected with the Arrb1-HA, Gli1-Flag and p300-HA plasmids, alone or in combination. *Left panel*: whole cell extracts were immunoprecipitated with anti-Flag agarose beads and immunoblotted with anti-HA, anti-Flag and anti-Gli1-ac antibodies. As negative controls, beads were pre-blocked with excess Flag peptide (0.1 mg/ml). *Right panel*: 2,5% of the immunoprecipitated cell lysates (input) were immunoblotted with the indicated antibodies. These experiments show that Arrb1 forms a complex with p300 and Gli1. **f** Luciferase activity of Gli-responsive reporter (Gli8x_luc) in CSCs co-transfected with a wild-type Gli1-flag expression vector (Gli1 wt) or a Gli1K/R mutant (Gli1 K518R) together with the arrb1-HA or an empty vector as control. *p < 0.05 vs Empty; **p < 0.05 vs Gli1 wt. **g** *Left panel*: Oncosphere forming assay in CSCs after ectopic expression of Arrb1. Data represent mean ± S.D. from three independent experiments. *p < 0.05. *Right panel*: WB analysis of endogenous Nanog along with ectopic Arrb1 in CSCs overexpressing Arrb1 or empty vector as control (Ctr). LC: actin. **h** BrdU uptake in CSCs after ectopic expression of Arrb1. Data represent mean ± S.D. from three independent experiments. *p < 0.05

Interestingly, we observed that Gli1 protein levels sharply decreased in CSCs overexpressing Arrb1 (Fig. 4a) and in response to DFM (Fig. 4b). Notably, Gli1 downregulation was preceded by an early increase of its acetylated form (Fig. 4b and c).

These results show that shifting SHH-MB CSCs to DFM induces the expression of Arrb1 and is linked to Gli1 acetylation, a modification that limits the activity of this transcription factor [20]. Since Gli1 acetylation is regulated by p300 [20] we sought to investigate the role of Arrb1 in this regulatory mechanism. We discovered that Gli1 acetylation was strongly impaired by Arrb1 knockdown (Fig. 4d). Moreover we show that Arrb1 formed a complex with both p300 and Gli1 (Fig. 4e). To further link Arrb1 to Gli1 acetylation and activity, we tested the effects of exogenously expressed Arrb1 on transcriptional activation of a Gli-responsive luciferase reporter by wild-type Gli1 or the Gli1K518R acetylation defective mutant [20]. As shown in Fig. 4f, Arrb1 inhibited the activity of wild type Gli1, whereas the Gli1K518R mutant was not affected. Taken together, these results indicated that Arrb1 inhibits Hh/Gli signaling through the modulation of Gli1 K518 acetylation. Overexpression of Arrb1 in CSCs significantly reduced clonogenicity (Fig. 4g left), Nanog protein levels (Fig. 4g right) and cell proliferation rate (Fig. 4h).

Altogether these results show that miR-326 and Arrb1 inhibit CSCs self-renewal and proliferation by suppressing Hh/Gli signaling at multiple levels. Collectively, this data suggest that the previously described acetylation mechanism of Gli1 and Gli2 function [20] is a part of a regulatory Arrb1/p300-dependent circuitry in cancer context.

### miR-326, pri-miR-326 and ARRB1 are coherently down regulated in human SHH-MBs

**Fig. 5:**
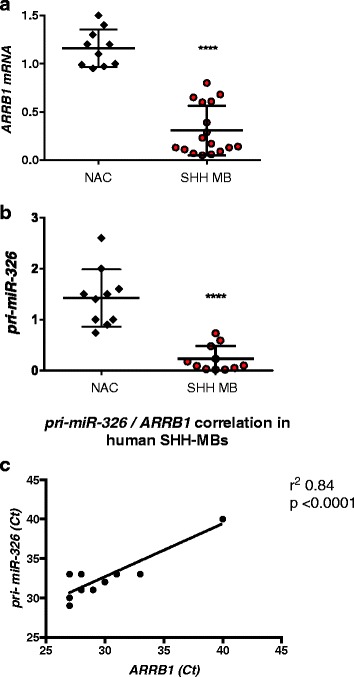
*ARRB1 and pri-miR-326 expression in human SHH-MB.* **a** Expression of ARRB1 in human primary SHH-MBs (*n* = 17), evaluate by qRT-PCR, compared to normal adult cerebella (NAC) ****p < 0.0001. **b** Expression of pri-miR-326 in human primary SHH-MBs, evaluated by qRT-PCR, compared to normal adult cerebella (NAC) ****p < 0.0001. **c** Linear Regression analysis of ARRB1 and pri-miR-326 levels in SHH-MB. Scatter plot show Spearman correlation ARRB1 and pri-miR-326 Ct values in each single SHH MB. r square = 0. 8424; ****p < 0.0001

We previously reported that human SHH-MB expressed low levels of mature miR-326 [23, 33]. Taking in consideration our results in mouse models of SHH-MB we evaluated *ARRB1* and *pri-miR-326* expression levels in primary tumors derived from SHH-MB patients. Here we found that *ARRB1* mRNA, as well as *pri-miR-326*, were significantly down-regulated in SHH-MBs when compared to adult cerebella (Fig. 5a and b) and their expression levels were highly correlated, as revealed by regression analysis (Fig. 5c).

Altogether these results showed that human SHH-MBs, characterized by an aberrant activation of the Hh/Gli signaling pathway, displayed a coordinated downregulation of ARRB1 and miR-326.

## Discussion

**Fig. 6:**
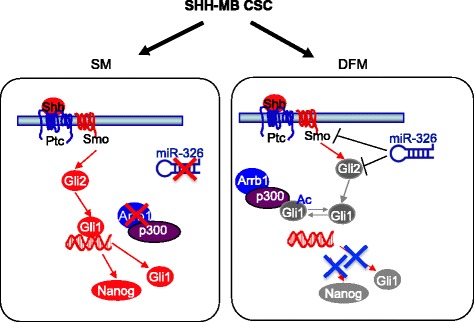
*Schematic overview of Arrb1 and miR-326 role in CSCs. Left*: Arrb1 and miR-326 expression are downregulated in CSCs derived from SHH-MB. These molecular features have a role in the maintenance of Hh/Gli signaling in CSCs. *Right*: Arrb1/miR-326 levels increase following exposure to differentiation signals (DFM) or after ectopic re-expression. Arrb1 protein enhances p300-induced acetylation of Gli1 (Gli-Ac) reducing its transcriptional activity. miR-326 targets and represses Smo and Gli2 mRNAs resulting in a blunted Hh/Gli signaling. The consequent low Hh/Gli signaling induces stemness impairment, growth arrest and facilitates differentiation of CSCs

We identified the down regulation of miR-326 and its host gene Arrb1 as a critical feature of CSCs derived from SHH-MB (Fig. 6). Re-expression of miR-326 and Arrb1 inhibits Hh/Gli pathway by targeting multiple activator components of this signaling Smo, Gli2 and Gli1 required for CSCs behavior. This conclusion is supported by at least two lines of evidence. First, low levels of miR-326 and Arrb1 characterized SHH-MB CSCs whereas CSCs in DFM re-express miR-326 and Arrb1 that leads to their differentiation and loss of stemness markers. Second, Arrb1 and miR-326 converge upon the Hh/Gli downstream signaling pathway that regulates cell growth and stemness. Indeed, we show that miR-326 suppresses Hh/Gli signaling by targeting Smo and Gli2, and that Arrb1 limits Gli1 transcription activity by promoting p300-dependent Gli1 acetylation further inhibiting Hh/Gli signaling.

miR-326 is a recognized tumor-suppressing miRNA, in fact among its targets there are Gli2, Smo, Notch1, Notch2 and Nob1 [23, 40–46]. miR-326 has been already described down regulated in several tumors [40–42], including brain tumor, e.g. medulloblastoma itself [23] and glioblastoma [43–45] where it targets key molecules of gliomagenesis [43, 46] and its ectopic expression impaired the viability of both glioma cell lines and glioma stem-like cells [43]. Of note, miR-326 levels have been shown to have a prognostic significance in glioblastoma patients [47].

In our study we focused on the role of miR-326 and its host gene Arrb1 in SHH-MB CSCs context. Previous studies have shown that Arrb1 functions as an adaptor/scaffold protein that shuttles between the cytoplasm and the nucleus, where it interacts with CREB and with p300 acetyltransferase on the promoters of target genes to enhance H3 and H4 histones acetylation and gene expression [48–51]. Our observation that Arrb1 promotes p300-mediated acetylation of Gli1 is consistent with such described ability to facilitate p300-dependent acetylation [31, 39] and extends to a more direct modality to modulate gene expression in the nucleus. We previously reported that Gli1 activity is regulated by acetylation [20, 32]. HDAC1-mediated deacetylation enhances Gli1 transcriptional activity, whereas acetylation at specific lysine residues inhibits their function [20, 32]. We show here that p300 is the HAT involved and, more importantly, that Arrb1 enhances this function. Thus, Arrb1 targets Gli1 and has a specific role in controlling Hh/Gli pathway and stemness. However we cannot exclude that such mechanism could involve other acetyl-transferases, e.g. p300/CBP-associated factor (PCAF) which has been reported to be able to interact with both Gli1 [52] and Arrb1 [53]. Similarly, the pleiotropic effect achievable by modulating protein acetylation may underlie other possible functions of Arrb1 in controlling other mechanisms of cell growth arrest [48].

Altogether our results indicate that miR-326 and Arrb1 encode a double signal (miRNA and hosting protein encoding gene) that cooperates to control SHH-MB, both in mouse models and in human MBs, through the modulation of morphogenetic signals Hh/Gli.

## Conclusion

In conclusion, in our study we identified a new molecular mechanism involving miR-326 and Arrb1 as regulators of SHH-MB CSCs. We demonstrated that low levels of miR-326 and Arrb1 trigger and sustain Hh/Gli signaling activation and self-renewal in SHH-MBs. The re-expression of both elements of this locus is able to impair self-renewal and proliferation of SHH-MB CSCs converging on blunting Hh signaling at multiple levels. These findings contribute to the understanding of the SHH-MB biology/behavior unveiling a mechanism underlying the cancer stem cell maintenance.

## Abbreviations

ARRB1: β-arrestin1 human
Arrb1: β-arrestin1 mouse
CSCs: Cancer stem cells
DFM: Differentiation medium
GCPs: Cerebellar granule cell progenitors
Gli1: Glioma associated oncogene homolog 1
Gli1-ac: Acetylated Gli1 form
Hh/Gli: Sonic Hedgehog/Gli
Ptch: Patched
SHH-MB: Sonic hedgehog medulloblastoma
SM: Selective medium
Smo: Smoothened
